# Interleaving cerebral CT perfusion with neck CT angiography part I. Proof of concept and accuracy of cerebral perfusion values

**DOI:** 10.1007/s00330-016-4577-y

**Published:** 2016-10-07

**Authors:** Marcel T. H. Oei, Frederick J. A. Meijer, Willem-Jan van der Woude, Ewoud J. Smit, Bram van Ginneken, Mathias Prokop, Rashindra Manniesing

**Affiliations:** Department of Radiology and Nuclear Medicine, Radboud University Medical Center, P.O. Box 9101, 6500 HB Nijmegen, The Netherlands

**Keywords:** Multidetector computed tomography, Angiography, Perfusion, Brain, Stroke

## Abstract

**Objectives:**

We present a novel One-Step-Stroke protocol for wide-detector CT scanners that interleaves cerebral CTP with volumetric neck CTA (vCTA). We evaluate whether the resulting time gap in CTP affects the accuracy of CTP values.

**Methods:**

Cerebral CTP maps were retrospectively obtained from 20 patients with suspicion of acute ischemic stroke and served as the reference standard. To simulate a 4 s gap for interleaving CTP with vCTA, we eliminated one acquisition at various time points of CTP starting from the bolus-arrival-time(BAT). Optimal timing of the vCTA was evaluated. At the time point with least errors, we evaluated elimination of a second time point (6 s gap).

**Results:**

Mean absolute percentage errors of all perfusion values remained below 10 % in all patients when eliminating any one time point in the CTP sequence starting from the BAT. Acquiring the vCTA 2 s after reaching a threshold of 70HU resulted in the lowest errors (mean <3.0 %). Eliminating a second time point still resulted in mean errors <3.5 %. CBF/CBV showed no significant differences in perfusion values except MTT. However, the percentage errors were always below 10 % compared to the original protocol.

**Conclusion:**

Interleaving cerebral CTP with neck CTA is feasible with minor effects on the perfusion values.

***Key Points*:**

• *Removing a single CTP acquisition has minor effects on calculated perfusion values*

• *Calculated perfusion values errors depend on timing of skipping a CTP acquisition*

• *Qualitative evaluation of CTP was not influenced by removing two time points*

• *Neck CTA is optimally timed in the upslope of arterial enhancement*

**Electronic supplementary material:**

The online version of this article (doi:10.1007/s00330-016-4577-y) contains supplementary material, which is available to authorized users.

## Introduction

A separate head and neck CT angiography (CTA) and cerebral CT perfusion (CTP) acquisition in addition to cerebral non-contrast CT (NCCT) is commonly performed in the diagnostic workup of patients presenting with acute ischemic stroke [1–4]. This implies that the combination of CTA and CTP requires two doses of contrast agent. Since CTP can be used to provide excellent cerebral CTA as well [5–7], the technique can be optimized by only covering the region of the neck vessels in a separate CTA acquisition [8].

If the cerebral CTP acquisition sequence could be interleaved with the neck CTA acquisition, no additional contrast injection would be necessary and the total exam time would be reduced in a clinical setting where rapid treatment decisions are of paramount importance. We present a novel scanning technique for wide-detector CT scanners that obviates the need for a separate head and neck CTA acquisition, which we therefore consider a *One-Step-Stroke* protocol. This technique relies on a wide detector coverage and a rapid table movement to interleave a whole brain CTP with neck CTA in one sequence using a single dose of contrast agent. The sequence begins with CTP acquisitions of the brain. At a suitable time point, a volumetric neck CTA is acquired by rapidly moving the table to the adjacent neck region. Then, the table moves back to the brain, where the CTP acquisition is continued. By using a single exam imaging time, radiation exposure and amount of contrast material can be reduced. A prerequisite for this technique is that the perfusion maps resulting from such an interleaved sequence remain diagnostic. Ideally, the quantitative perfusion values should remain unaffected by the time gap introduced by performing the neck CTA within the CTP sequence.

The purpose of this study is to evaluate whether the resulting time gap in the CTP sequence affects the accuracy of the perfusion values.

## Materials and methods

Two authors (R.M. and M.P.) received a research grant from Toshiba Medical Systems Corporation (TMSC, Japan). Toshiba Medical Systems Corporation did not have any influence on the concept of the One-Step-Stroke protocol, the execution of this study, the analysis of the data, nor on the writing of this manuscript.

### Patient group

This retrospective study was approved by the ethics committee of our institution, and informed consent was waived. Initially, 34 consecutive subjects were included who underwent CTP scanning at the emergency department of our hospital. Inclusion criteria were: patients with clinical symptoms of acute ischemic stroke, with onset of symptoms within 9 hours, without a history of kidney failure and a minimum age of 18 years. Exclusion criteria were: non-standard CTP acquisition protocol (4), severe patient movement artefacts in CTP (2), incidental finding of a tumour lesion (1), late or poor contrast enhancement (3), intra-arterial contrast injection (1), the presence of a drainage tube (1), clipped or coiled cerebral aneurysm (2). The remaining 20 patients consisted of eight male and 12 female patients (mean age 65 years, median 66 years and age range 36 - 93 years).

In eight out of 20 patients, signs of acute ischemic stroke were seen on the NCCT, CTA and/or CTP images by the attending neuroradiologist.

### CTP protocol

CT imaging was performed on a 320-row CT scanner (Toshiba, Aquilion ONE, Toshiba Medical Systems Corporation, TMSC, Otawara, Japan). The scan protocol consisted of a cerebral NCCT, cerebral CTP, and head and neck CTA. In all patients two contrast injections were performed, one for CTP and one for the CTA. Only the CTP acquisitions were used in the present study.

**Fig. 1:**
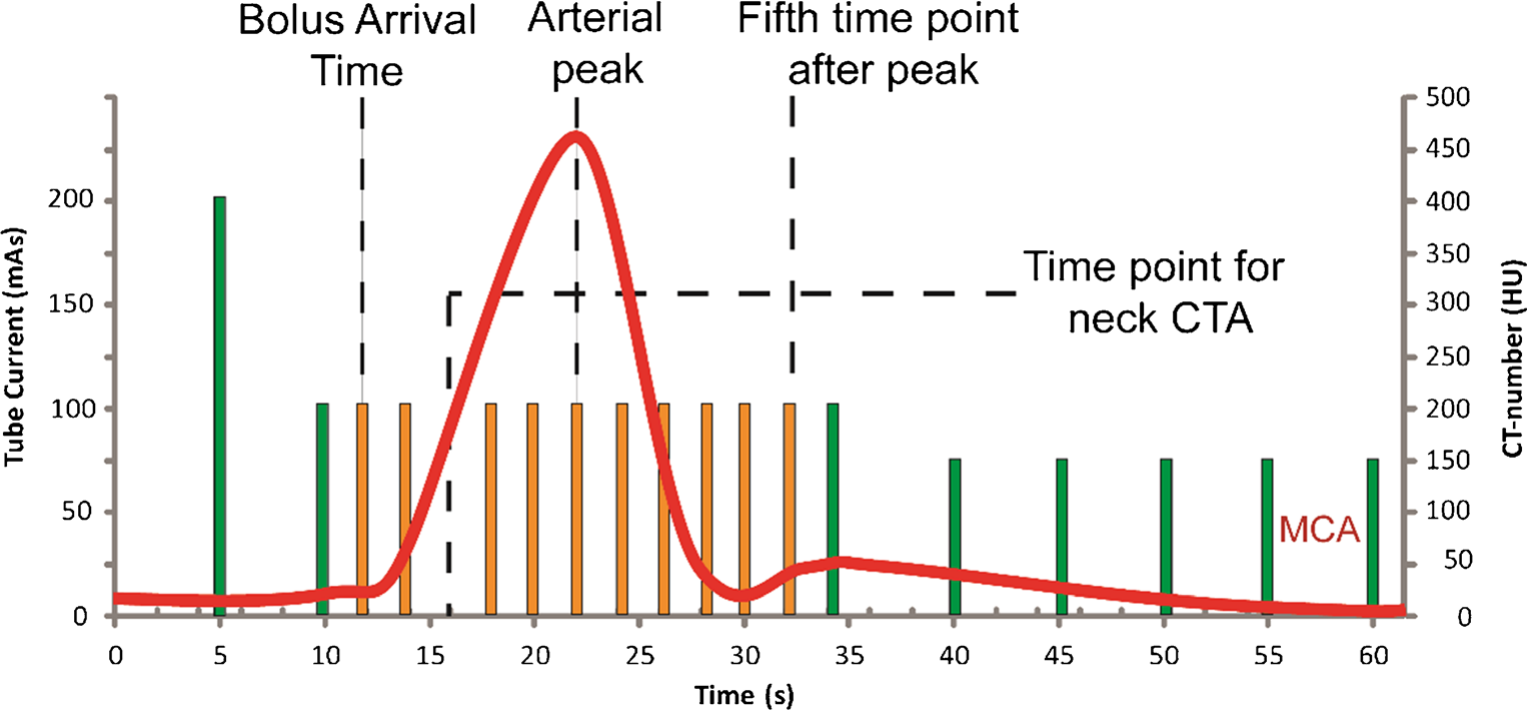
Graphical overview of the clinical CTP protocol. The vertical bars denote the individual time points of the protocol; the height of each bar represents the tube current at that time point. The red line is the attenuation curve in the middle cerebral artery in the proximal M1 segment (MCA). For every patient bolus arrival time and peak enhancement in the MCA were determined. The One-Step-Stroke protocol was simulated by eliminating one volumetric acquisition starting from the bolus arrival time up to the fifth time point after peak enhancement of the M1-MCA. These are denoted by the orange bars

For CTP, 50 mL nonionic contrast agent (300 mg iodine/mL Xenetix 300, Guerbet, Villepinte, France) was injected into an antecubital vein with an injection rate of 5 mL/s followed by a 40 mL saline flush at 5 mL/s. Whole brain volumetric acquisitions with 16 cm z-coverage were acquired with 0.5 mm slice thickness, 0.5 s rotation time, and 80 kV tube voltage. A cerebral CTP protocol was used, which started 5 s after contrast agent injection with the first volumetric acquisition at 200 mAs, followed after 4 s by 13 scans at 100 mAs with a 2 s interval, followed after 5 s by five scans at 75 mAs with a 5 s interval. The total number of scans was 19 and total scan duration was 60 s (Fig. 1). Image reconstruction was done using a smooth convolution kernel FC41 and standard AIDR3D (adaptive iterative dose reduction in three-dimensions, TMSC).

### Perfusion analysis

A publicly available software program, Perfusion Mismatch Analyzer (PMA) developed by the Acute Stroke Imaging Standardization Group (ASIST), version 5.0.0.0, was used to calculate perfusion maps of cerebral blood flow (CBF), cerebral blood volume (CBV), and mean transit time (MTT). The software automatically selects ten arterial input functions (AIFs) in one slice, which was set at the level of the Circle of Willis. The venous output function (VOF) was automatically chosen in the intracranial veins above the skull base. The perfusion maps were calculated with a delay-insensitive deconvolution algorithm, the block-circulant Singular Value Decomposition (bSVD) [9]. Calculations were performed on a 256 × 256 matrix with smoothing enabled, on 5 mm slabs of the original CTP data; other parameters of PMA were kept at default values.

### Simulating the one-step-stroke protocol

The One-Step-Stroke protocol was simulated by eliminating specific acquisitions from a CTP sequence in order to study the optimal time gaps between subsequent acquisitions, similar to a previous study published in the literature [10]. The perfusion values derived from the original sequence served as the reference standard. One observer experienced in CTP analyses (M.O.) selected a region of interest (ROI) in the (unaffected) proximal M1-portion of the middle cerebral artery (M1-MCA) to visually determine the bolus arrival time (BAT). For every patient one volumetric acquisition was deleted, starting from the BAT up to the fifth time point after the arterial peak (see Fig. 1). Because the number of volumetric acquisitions between these two markers may differ per patient, the number of deleted time points and, therefore, the number of simulated One-Step-Stroke protocols differ per patient. Perfusion maps were calculated for the original CTP acquisition and for each One-Step-Stroke protocol. The locations of the automatically chosen AIF and VOF of these simulated One-Step-Stroke protocols were compared to the locations in the original protocol and manually corrected if they were not at identical locations. The ROI in the MCA was also used to estimate arterial enhancement to estimate the enhancement of the carotid arteries.

### Data analysis

Perfusion values in normal-appearing white matter (WM) and normal-appearing gray matter in the basal ganglia (GM) were estimated by drawing multiple ROIs (M.O.) and averaging perfusion values across these ROIs for each patient and tissue type. White matter ROI’s included the centrum semiovale and cortical spinal tract. Subcortical gray matter ROI’s included the caudate nucleus, putamen and globus pallidus. The size and location of the ROIs were kept constant within the patient, while the size of the ROI’s differed between patients (freehand ROI’s were applied). The total number of voxels included was 6,922 ± 3,674 (mean ± standard deviation) for NAWM and 547 ± 244 for NABG.

For each skipped time point, percentage errors of the perfusion values were calculated per tissue type and patient. The percentage error was calculated by taking the difference of the perfusion values between the original CTP protocol and the simulated One-Step-Stroke protocol divided by the perfusion value of the original CTP protocol multiplied by 100. Since these percentage errors can be positive or negative, we used the absolute percentage error |% error| for further analysis.

First, to estimate the magnitude of the absolute percentage errors, we determined the mean and standard deviation of all patients and all simulated One-Step-Stroke protocols, for CBV, CBF, and MTT in WM and GM. Thus, in total six mean absolute percentage errors and corresponding standard deviations were calculated. Furthermore, we report the maximum absolute percentage errors.

Next, we determined the optimal timing of the neck CTA. Since we aimed to evaluate which time point could be deleted without having a major influence on any of the three perfusion parameters, for each patient we selected the maximum percentage error across the three perfusion parameters CBV, CBF, and MTT in WM and GM per deleted time point. Thus, per patient and per simulated One-Step-Stroke protocol (i.e., per deleted time point), one maximum percentage error is selected for further analyses.

In order to determine the optimal timing of the neck CTA, we simulated bolus tracking by determining the first time point T_0_ in which the enhancement in the M1-MCA exceeded a given relative threshold value. Relative thresholds, i.e., enhancement above the baseline pre-contrast scan, were varied between 40 HU and 100 HU in steps of 10 HU. Every deleted time point was reported in seconds relative to T_0_, for up to 10 s after T_0_.

Therefore, each threshold defines a T_0_ (which may differ per patient in absolute time) and each simulated One-Step-Stroke protocol results in a maximum percentage error per patient. The mean (maximum percentage error) of all patients was then calculated. We calculated the mean maximum percentage error as a function of the relative threshold (a trigger for bolus tracking) and as a function of the deleted time points from T_0_.

To estimate how often the selected timing would lead to larger errors, we reported the number of patients in whom the absolute percentage error exceeded 10 % in at least one perfusion parameter. The time point and threshold with the lowest average maximum percentage error and the lowest number of patients with more than 10 % errors in combination with the highest enhancement in the MCA (which served as proxy for carotid enhancement) was chosen as the optimal timing.

Finally, after determining the optimal time point for neck CTA the analysis was repeated but with two time points deleted to simulate a 6 s gap at the optimal timing only.

### Visual assessment of the perfusion maps

A subsequent observer study was performed in order to evaluate the influence of skipping two CT perfusion time points on the qualitative evaluation of perfusion maps. An experienced observer (F.J.A.M.) evaluated the original perfusion maps and perfusion maps with a 6 s time gap randomly. The CT perfusion maps were scored for: 1) The presence or absence of a perfusion deficit. 2) Absence or presence of infarct core. 3) Absence or presence of penumbra. and 4) Size of infarct core or penumbra. The sizes were described as either small or large relative to the vascular territory affected. Infarct core was defined by a perfusion deficit with increased MTT, decreased CBF, and decreased CBV. Penumbra was defined as the presence of a perfusion deficit in relation with normal or slight elevated CBV. The observer was blinded to perfusion maps shown (original or with a 6 s gap), clinical information and diagnoses.

### Statistical analysis

Statistical analyses were performed using the Statistical Package of Social Sciences version 20.0 for Windows (SPSS Inc., Chicago, USA). A Wilcoxon signed rank test was used to show significant differences between the original CTP protocol and the One-Step-Stroke protocol at the optimal time point with 4 s and 6 s gaps. A *P* value < 0.05 was considered significant. By examining the slope of a linear fit intersecting the origin, the linear relationship between the original CTP protocol and the One-Step-Stroke protocol with 4 s and 6 s gaps were assessed. A linear fit of 1 was considered ideal. Spearman correlation coefficients were reported. Bland-Altman analyses were performed to compare the original CTP and the simulated One-Step-Stroke with 4 s and 6 s time gaps.

## Results

### Absolute percentage errors

Table 1 gives an overview of the absolute percentage errors. In WM, the mean absolute percentage errors were 3.1 % for CBF, 2.7 % for CBV, and 1.6 % for MTT. In GM, we found an average absolute percentage error of 3.5 % for CBF, 2.8 % for CBV, and 2.6 % for MTT of all patients and time points.

**Table 1:** Mean, standard deviation and maximum absolute percentage errors

	White Matter	Gray Matter
|% error| CBF	3.1 ± 3.7 (22.2)	3.5 ± 3.5 (30.1)
|% error| CBV	2.7 ± 3.1 (18.5)	2.8 ± 3.2 (24.6)
|% error| MTT	1.6 ± 1.4 (7.4)	2.6 ± 2.4 (13.8)

Note - Absolute percentage error of CT perfusion values in white matter and gray matter if one time point of the CTP sequence is skipped, averaged for all patients and for all deleted time points. Values shown are the mean ± standard deviations and maximum absolute percentage error in parentheses. Note that despite a low mean across all time points and patients, the maximum error may be substantial

However, the maximum errors that could occur were substantially larger: 22.2 % for CBF, 18.5 % for CBV, and 7.4 % for MTT in WM, and 30.1 % for CBF, 24.6 % for CBV, and 13.8 % for MTT in GM. The absolute percentage errors exceeded 10 % in 16 instances in CBF (seven in GM and nine in WM), 14 instances in CBV (five in GM and nine in WM), and two instances in MTT (GM).

Supplementary Figure 4 gives an example of a patient in whom the percentage errors exceeded 10 % in all perfusion maps if an unsuitable time point was chosen.

### Optimal timing

The results of optimization are summarized in Tables 2 and 3. Table 2 displays the mean maximum absolute percentage error across all perfusion parameters, and provides the number of patients in whom the absolute percentage error exceeded 10 %. Given a threshold in the range of 40–70 HU, the percentage errors never exceeded 10 % if the neck CTA was acquired 2 s after the first scan in which enhancement exceeded the threshold. A relative threshold of 70 HU gave the lowest percentage errors.

**Table 2:** Optimization of timing and threshold for bolus tracking

	40 HU	50 HU	60 HU	70 HU	80 HU	90 HU	100 HU
T_0 +_ 2 s	4.1 ± 2.1 (1.7 – 9.5) *n* = 0	4.0 ± 2.8 (1.7 – 9.5) *n* = 0	3.9 ± 1.8 (1.9 – 7.5) *n* = 0		4.2 ± 2.5 (1.9 – 12.0) *n* = 1	4.5 ± 2.6 (1.9 – 12.0) *n* = 1	5.4 ± 4.7 (1.9 – 22.0) *n* = 2
T_0 +_ 4 s	4.2 ± 2.8 (1.1 – 12.0) *n* = 1	5.5 ± 4.8 (1.1 – 22.0) *n* = 2	5.3 ± 4.9 (1.1 – 22.0) *n* = 2	5.3 ± 4.9 (1.1 – 22.0) *n* = 2	4.8 ± 4.7 (1.1 – 22.0) *n* = 1	4.6 ± 4.6 (1.1 – 22.0) *n* = 1	4.4 ± 4.3 (1.1 – 20.5) *n* = 1
T_0 +_ 6 s	4.5 ± 4.5 (1.3 – 22.0) *n* = 1	4.8 ± 4.7 (1.3 – 20.5) *n* = 2	4.9 ± 4.6 (1.7 – 20.5) *n* = 2	4.9 ± 4.6 (1.1 – 20.5) *n* = 2	5.3 ± 4.5 (1.9 – 20.5) *n* = 2	5.4 ± 4.5 (1.9 – 20.5) *n* = 2	6.3 ± 6.4 (1.9 – 30.1) *n* = 3
T_0 +_ 8 s	6.2 ± 4.5 (1.9 – 20.5) *n* = 3	6.2 ± 5.9 (1.9 – 30.1) *n* = 2	6.9 ± 6.2 (1.6 – 30.1) *n* = 3	7.0 ± 6.2 (1.6 – 30.1) *n* = 3	7.1 ± 6.3 (1.6 – 30.1) *n* = 3	7.1 ± 6.3 (1.6 – 30.1) *n* = 3	5.2 ± 3.1 (1.6 – 15.8) *n* = 1
T_0 +_ 10s	6.2 ± 6.5 (1.6 – 30.1) *n* = 2	4.6 ± 3.5 (1.2 – 15.8) *n* = 1	4.4 ± 2.6 (1.2 – 9.7) *n* = 0	4.3 ± 2.7 (1.2 – 9.7) *n* = 0	4.2 ± 2.4 (1.2 – 9.7) *n* = 0	4.0 ± 2.4 (1.2 – 9.7) *n* = 0	5.2 ± 4.7 (1.2 – 22.2) *n* = 1

Note - For each patient the maximum of the errors of CBV, CBF and MTT was calculated for each combination of enhancement threshold (horizontally) and post-threshold delay (vertically). The table displays the mean ± standard deviation and the range for each combination averaged over all patients. In addition, the number of patients in whom the maximum error exceeded 10 % is listed. Note that the lowest error occurred for a threshold of 70 HU above baseline (green box), which is selected as the optimal timing (one deleted time point, 4 s time gap)

**Table 3:** Absolute percentage error of the perfusion values at the optimal timing

	Optimal Time with 4 s Gap		Optimal Time with 6 s Gap	
	White Matter	Gray Matter	White Matter	Gray Matter
|% error| CBF	2.0 ± 1.5 (5.5)	3.0 ± 2.1 (7.5)	3.5 ± 3.5 (13.9)	3.2 ± 2.3 (8.1)
|% error| CBV	1.6 ± 1.3 (5.7)	1.9 ± 1.7 (7.2)	3.4 ± 2.4 (9.1)	2.8 ± 2.2 (8.1)
|% error| MTT	1.6 ± 1.6 (5.2)	2.0 ± 1.2 (4.3)	2.9 ± 2.8 (9.2)	2.4 ± 1.9 (6.2)

Note – Data are shown in mean ± standard deviation, with the maximum value in parentheses. The mean across all patients remains low, and the maximum error is ≤7.5 % at optimal time with a 4 s gap and ≤9.2 % at optimal time with a 6 s gap (except for one patient for whom the CBF reached 13.9 %)

Table 3 provides the absolute percentage errors for CBV, CBF, and MTT for the case of optimal timing with a 4 s gap and with a 6 s gap. For optimal timing with a 4 s gap, all errors were below 7.5 %. At that time point, the average HU value in the M1 segment of the MCA were 302 ± 57.4 HU (range, 198 – 408 HU). For optimal timing with a 6 s gap, all errors were below 9.2 %, except for one patient for which CBF showed an error 13.9 %. On average, the percentage errors were below 3.5 %.

**Fig. 2:**
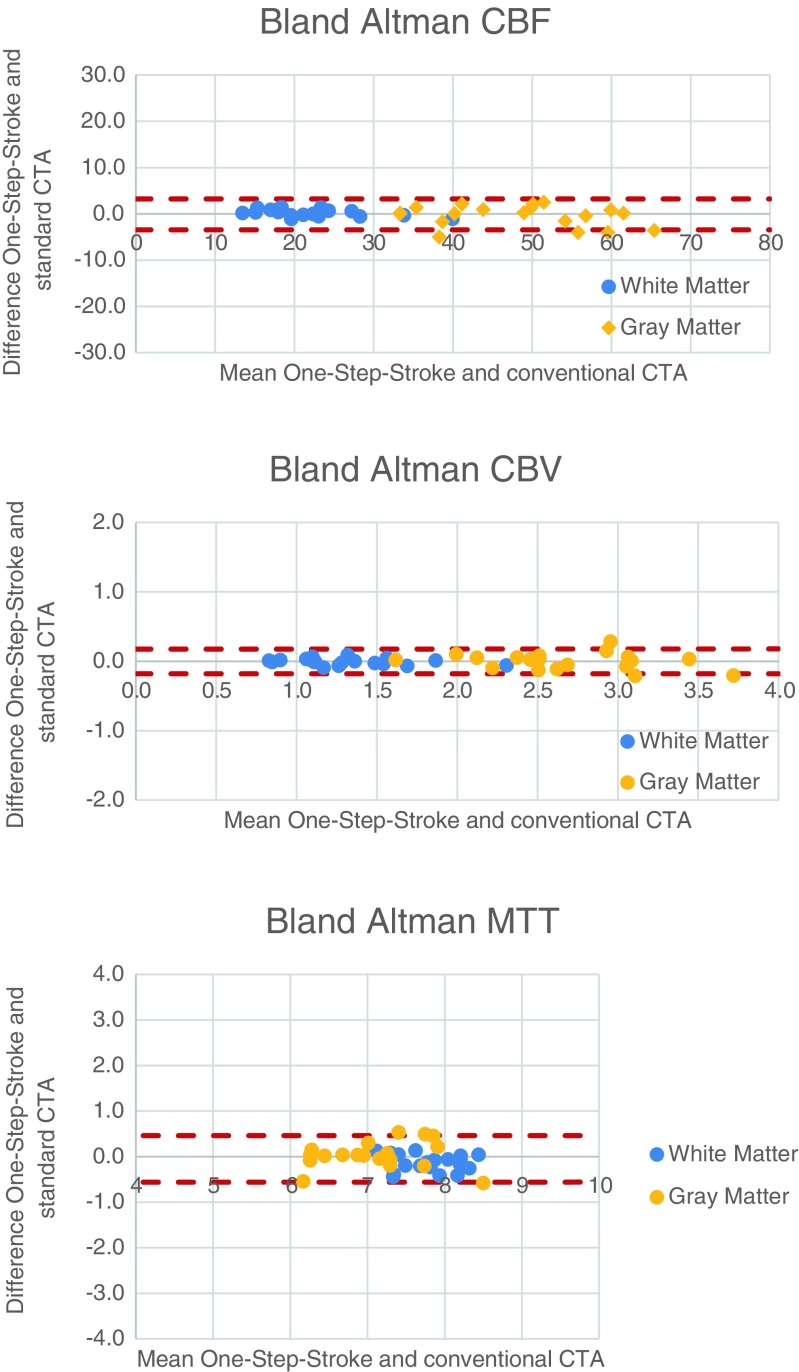
Bland Altman plots showing the perfusion values (CBF, CBV and MTT) between the original CTP protocol (the reference standard) and the One-Step-Stroke protocol 6 s time gap

Bland-Altman plots are shown in Fig. 2. Spearman correlations are shown in Table 4. There was no significant difference between the perfusion values of the One-Step-Stroke protocol at the optimal time point and the original CTP protocol, for both 4 s and 6 s time gaps, except for MTT in WM (see Table 4). Although MTT in WM showed a significant difference in perfusion values, the absolute perfusion values stayed within the 10 % (9.2 % for a 6 s time gap, and even 5.2 % for a 4 s time gap) compared to the original protocol.

**Table 4:** Comparison of perfusion values of the One-Step-Stroke Protocol at optimal time with a 4 s gap with the original protocol

	Optimal Time with 4 s Gap			Optimal Time with 6 s Gap		
	Spearman correlation		Wilcoxon signed ranked test	Spearman correlation		Wilcoxon signed ranked test
	*R*	*P* value	*P* value	*R*	*P* value	*P* value
White Matter						
CBF	.991	< .0001	.225	.971	< .0001	.232
CBV	.998	< .0001	.644	.973	< .0001	.808
MTT	.963	< .0001	**.011**	.907	< .0001	**.023**
Gray Matter						
CBF	.979	< .0001	.737	.961	< .0001	.940
CBV	.995	< .0001	.647	.944	< .0001	.723
MTT	.986	< .0001	.668	.913	< .0001	.765

Note – All correlations were found to be significant. Wilcoxon signed ranked test showed no significant differences between the means of the One-Step-Stroke protocol and the original protocol except for MTT in white matter. Despite this significance, the percentage errors remained below 9.2 % for a 6 s time gap, and even 5.2 % for a 4 s time gap (see Table 3)

### Visual assessment of the perfusion maps

In eight of 20 patients, a perfusion deficit was visible. All perfusion deficits were visible in the middle cerebral artery (MCA) territory. Two patients showed an infarct core of one third of the MCA territory with no penumbra and two patients showed small infarct core lesions in less than one third of the MCA territory with no penumbra. Two patients showed only penumbra of less than one third of the MCA territory. Two patients showed only penumbra which was more than two thirds of the MCA territory.

**Fig. 3:**
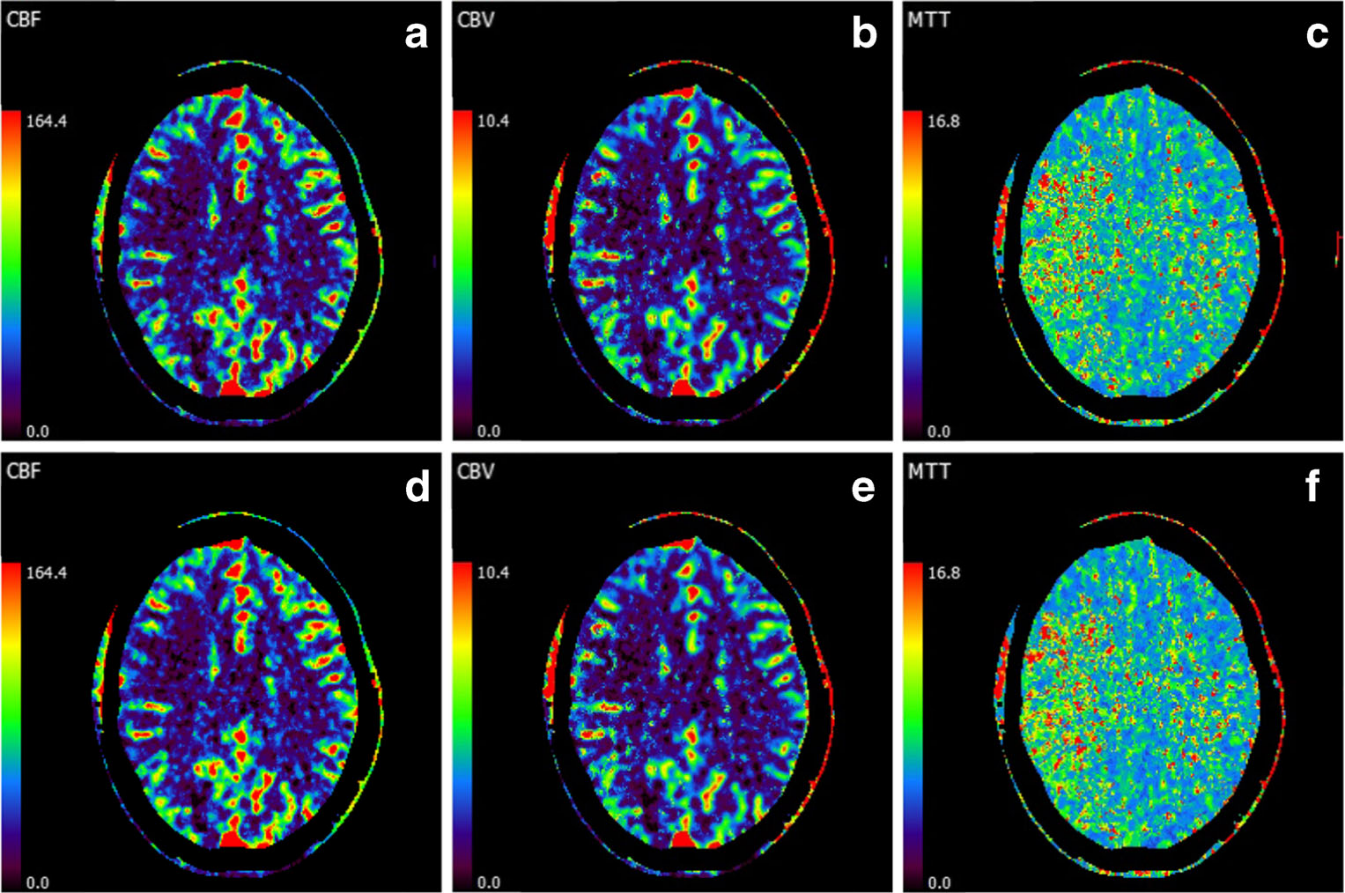
CT perfusion maps of a 41-year-old female with an infarct in the right MCA territory presented with weakness in left arm and legs and a right-sided face droop. In the upper row perfusion maps of the original CTP protocol are shown (**a**, **b**, and **c**). The lower row (**d**, **e**, and **f**) shows perfusion maps of the same patient in which the second time point was deleted. The perfusion images show an increased MTT with a small area of reduced CBV and CBF in the right MCA territory. Note that the perfusion maps are similar of the original and the perfusion maps with a 6 s gap. WL settings were not changed since the observer was shown the images as the perfusion software calculated the perfusion maps with these WL settings

The study showed full agreement between the original perfusion maps and the perfusion maps with a 6 s time gap in the detection of a perfusion deficit. However, in one case a possible small cortical perfusion deficit was noted in the original perfusion maps, while not rated in the perfusion maps with a 6 s gap; after reviewing the images next to each other, this was rather due to observer variability than to the imaging technique. At follow-up, no infarct was demonstrated in this area. No perfusion deficits were missed and the sizes of penumbra and infarct core were described similar in both perfusion maps. An example is shown in Fig. 3.

## Discussion

Our study shows that a One-Step Stroke protocol is feasible with only minor differences in CTP values and good arterial enhancement for the neck CTA, if a suitable time point is used for skipping one volumetric CTP acquisition and performing the neck CTA instead. In fact, the absolute percentage errors for CBV, CBF or MTT then never exceeded 7.5 %. Eliminating a second time point still resulted in average errors <3.5 % and maximum percentage errors <10 %. Arterial enhancement in the MCA was on average above 300 HU if the neck CTA was performed 2 s after a threshold of 70 HU was reached in the MCA. Given that the timing is in the upslope of the arterial enhancement curve, the enhancement in the carotid artery can be expected to be even higher because the enhancement in the MCA tails enhancement in the carotids.

Generally, perfusion values have high variability due to differences in, e.g., acquisition protocol, post-processing software and the manual selection of AIF and VOF [11]. A previous study assessed the effect of manually outlining ROIs in various flow territories and found that the observer variability for perfusion values was 11-18 % for CBV, 15-19 % for CBF, and 6-9 % for MTT [12]. Another study showed that the inter-observer variability of perfusion values resulting from selection of AIF and VOF was 27.1 % for manual selection and 10.4 % for automatic selection [13]. Two studies reported that it is unlikely that clinical decisions alter with qualitative visual assessment of the perfusion maps, if an overall variability around 10 % is achieved [14, 15]. We, therefore, chose a 10 % limit for absolute percentage errors as an indicator of errors that might become clinically relevant. With the optimal timing suggested in this article, the errors always remain below 7.5 %.

A comparable study showed that CTP with a 3 s (if 50 ml contrast agent was given) or 4 s (if 60 ml contrast agent was given) temporal sampling interval over the first 60 s does not significantly over- or underestimate the perfusion values relative to protocols with 1 s sampling intervals [10]. In concordance with their findings, the average absolute percentage errors reported in this study ranged between 1.6 % for MTT in WM and 3.5 % for CBF in GM across all time points and patients. We found, however, substantially higher errors in certain patients and time points that ranged between a maximum of 7.4 % for MTT in WM to 30.1 % for CBF in GM. We believe that such errors are not acceptable, if they can be avoided by an optimal timing of the excluded time point for the neck CTA. Nonetheless, even with maximum errors up to 17 %, the perfusion maps of the One-Step-Stroke protocol have strong visual resemblance with perfusion maps of the original protocol (Supplementary Fig. 4, see also Fig. 3), with persistence of relative differences in perfusion between white matter and gray matter.

In current literature, scan durations longer than 60 to 90 s are advised to avoid truncation of the tissue curves [16]. The scan duration of our CTP protocol is 60 s, which in our experience is sufficient for the enhancement of collateral vessels and to avoid truncation of the tissue curves which may affect perfusion values [7]. Our data verified that there was no truncation of the tissue curves in all patients.

Our study has some limitations. First, the sample size is relatively small. However, mean percentage errors remain small independent of timing, and in none of the 20 patients the error exceeded 7.5 % for the suggested timing. In addition, visual analysis on CTP maps will probably still provide sufficient information to detect cerebral perfusion deficits as relative differences in perfusion values are probably not affected. The observer study showed full agreement between the original perfusion maps and the perfusion maps with two missing time points. Second, only perfusion values of ROIs in normal appearing white matter and basal ganglia were investigated. In each patient we kept all ROIs constant during the experiments of removing one time point from the CTP dataset in order not to introduce another source of variation. We choose not to annotate infarct core or penumbra because of the subjectivity of determining those areas and because such areas were not present in all patients. Third, it is known that perfusion values are also dependent on the software package used [17, 18]. Therefore we used only one software package for evaluation, which is publicly available and vendor independent. We found that our average percentage errors are consistent with values reported by another study [10] who used a different software package.

In conclusion, our study showed that the One-Step-Stroke protocol has only minor effects on the calculated perfusion values, if the neck CTA is acquired 2 s after a relative threshold of 70 HU is observed in the MCA. Visual assessment of the perfusion maps with a 6 s gap did not affect the detection of a perfusion deficit.

## Electronic supplementary material

Below is the link to the electronic supplementary material.

Supplementary Figure 4 (DOCX 452 kb)
