# Supplementary material for: Interleaving cerebral CT perfusion with neck CT angiography part I. Proof of concept and accuracy of cerebral perfusion values
Source: Eur Radiol. 2016 Oct 7;27(6):2649–56. doi: 10.1007/s00330-016-4577-y (PMC5409805; doi:10.1007/s00330-016-4577-y)
Supplement: Supplementary file 1 — (DOCX 452 kb) [file 330_2016_4577_MOESM1_ESM.docx]

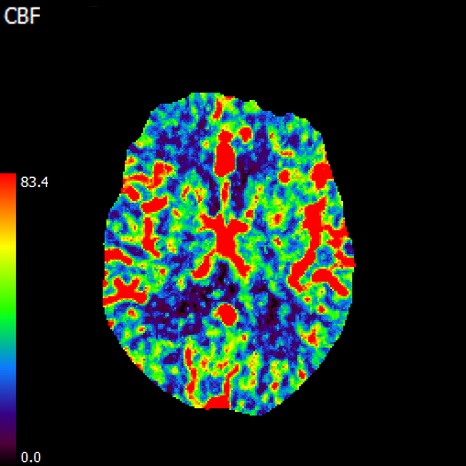

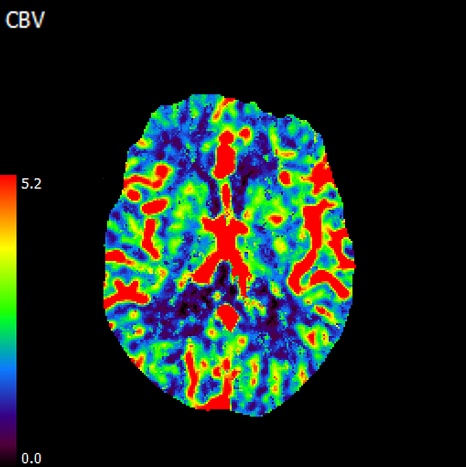

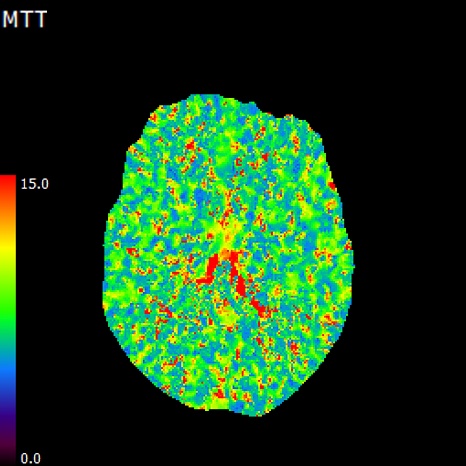


**A B C**


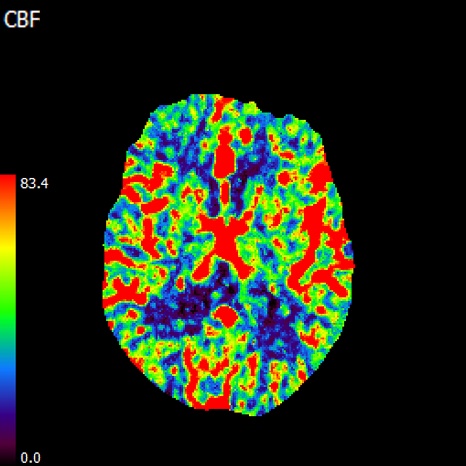

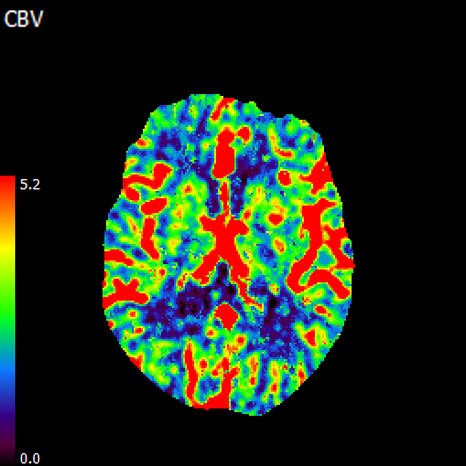

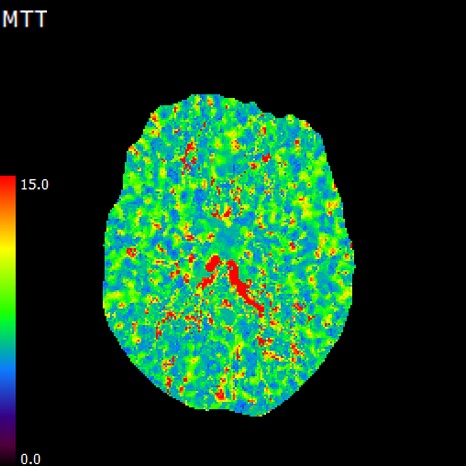


**D E F**

Supplementary Figure 4**:** CT perfusion maps of a 85 year old female with aphasia and a left-sided face droop. In the upper row perfusion maps of the original CTP protocol are shown (A, B, and C). The lower row (D, E, and F) shows perfusion maps of the same patient in which the second time point after arterial peak enhancement was deleted. Percentage errors varied between 10 and 17% for CBV and CBF measurements in white matter and gray matter. Errors in MTT varied between 4 and 7%. Note the subtle differences in color of the perfusion maps but the persistence of the relative differences in white and gray matter.
